# A Narrative Review of Heavy Metals and Sperm Quality: The Interplay with Antioxidant Imbalance and Reactive Oxygen Species

**DOI:** 10.3390/cimb47080650

**Published:** 2025-08-13

**Authors:** Soukaina Azil, Khaoula Errafii, Moncef Benkhalifa, Noureddine Louanjli, Bouchra Ghazi, Salsabil Hamdi

**Affiliations:** 1 Immunopathology-Immunotherapy-Immunomonitoring Laboratory, Faculty of Medicine, Mohammed VI University of Sciences and Health, Casablanca 82403, Morocco; bghazi@um6ss.ma; 2 Environmental Health Laboratory, Institut Pasteur du Maroc, Casablanca 20360, Morocco; 3 Laboratory of Medical Analysis and Reproductive Biology, Labomac, Casablanca 20250, Morocco; nlouanjli@gmail.com; 4 African Genomic Center (AGC), University Mohamed VI Polytechnic, Bengurir 43150, Morocco; khaoula.errafii@um6p.ma; 5 Reproductive Medicine, Reproductive Biology and Genetics, Peritox Laboratory, University Hospital and School of Medicine, Picardie University Jules Verne Amiens, 80054 Amiens, France; benkhalifa.moncef@chu-amiens.fr; 6 IVF Laboratory, Department of Reproductive Medicine, Mohammed VI International University Hospital, Bouskoura 27182, Morocco; 7 Mohammed VI Center for Research and Innovation (CM6RI), Rabat 10000, Morocco

**Keywords:** heavy metals, reproductive toxicity, male infertility, oxidative stress

## Abstract

Reproductive infertility is characterized by the inability to achieve pregnancy after a year or more of unprotected sexual intercourse. This review highlights the significant impact of exposure to both types of heavy metals (essential and non-essential) on the reproductive performance of various species, particularly humans. Heavy metals present a high atomic density and weight, including lead, mercury, cadmium, nickel, chromium, and arsenic, and are delivered into the environment through natural and human activities, posing a threat to ecological systems and human reproductive health. These heavy metals have the potential for bioaccumulation and can adversely affect male fertility and sperm quality due to their role in disrupting endocrine functions, altering hormone levels responsible for sperm production, and inducing oxidative stress. The elevated production of reactive oxygen species (ROS) exceeds the capability of antioxidants and can lead to the alteration of sperm quality. Seminal fluid contains antioxidants like vitamin C, vitamin E, zinc, and selenium to counteract the impacts of ROS and also to preserve the sperm function. This review aims also to explore the impact of heavy metals on sperm quality and their relationship with antioxidant imbalance and ROS. The exposure to heavy metals whether through occupational or environmental means increases the production of ROS and therefore leads to an imbalance of antioxidants production. All these factors have no doubt an impact on male reproductive health.

## 1. Introduction

Male fertility depends on the proper functioning of the hypothalamic–pituitary–gonadal axis and the integrity of testicular tissue, particularly the seminiferous tubules where spermatogenesis occurs [1]. This process involves the continuous division and maturation of germ cells, supported by Sertoli cells providing nutrients and coordinating germ cell differentiation. Leydig cells, located in the interstitial space, produce testosterone in response to luteinizing hormone, which is essential for the initiation and maintenance of spermatogenesis. Together, these cellular interactions maintain sperm production and hormonal balance. Disruption of these pathways can impair sperm quality, making them vulnerable to toxic agents such as heavy metals [2,3].

Heavy metals are ubiquitous environmental toxic substances and considered a serious danger to the ecological system, especially to the sexual and reproductive health of humans [4,5,6]. Heavy metals have a higher atomic density and weight, and among them we have lead (Pb), mercury (Hg), cadmium (Cd), nickel (Ni), chromium (Cr), copper (Cu), silver (Ag), iron (Fe), palladium (Pd), platinum (Pt), and arsenic (As). Due to the potential for bioaccumulation, the toxicity induced has become a major health concern given their effects on male fertility and impaired sperm quality [7,8]. On the other hand, seminal plasma presents some antioxidants and vitamins, for example, vitamin C and vitamin E, with some minerals, especially zinc and selenium, playing an important role with ROS in the maintenance of sperm activity [9].

Several studies have demonstrated that exposure to heavy metals adversely affects male reproductive function. For instance, in animal models, cadmium exposure has been shown to induce testicular atrophy, disrupt the blood–testis barrier, and impair spermatogenesis through oxidative stress and apoptotic pathways. Similarly, mercury has been associated with decreased testosterone levels and altered sperm morphology in rats [10,11]. In human studies, occupational exposure to lead has been correlated with decreased sperm concentration, reduced motility, and increased DNA fragmentation [12,13].

In this review we will discuss the relationship between heavy metals and sperm quality and their effect on the imbalance of antioxidants and ROS.

## 2. Heavy Metals

All species, especially humans, are exposed to heavy metals from multiple sources. Almost all food products are constantly made up of essential and non-essential materials due to the excessive use of agrochemicals and wastewater [14]. Arsenic, lead, and cadmium are detected in the environment, and are related to working conditions, occupations, and professional status, as well as the geographical location of industries, impacting the well-being of workers [15]. Several types of cancer have been a consequence of chronic exposure to heavy metals. Taking Marsh et al. as an example, they have shown the danger of nasopharyngeal cancer associated with work in the metal industry. A total of 7345 workers hired in a plastics production plant (1941–1984) in Wallingford were independently studied and evaluated as part of a National Cancer Institute cohort study. Up to 2003, they measured the vital status of 98% of the cohort, and the cause of death of 95% of the 2872 deaths [16].

**Figure 1:**
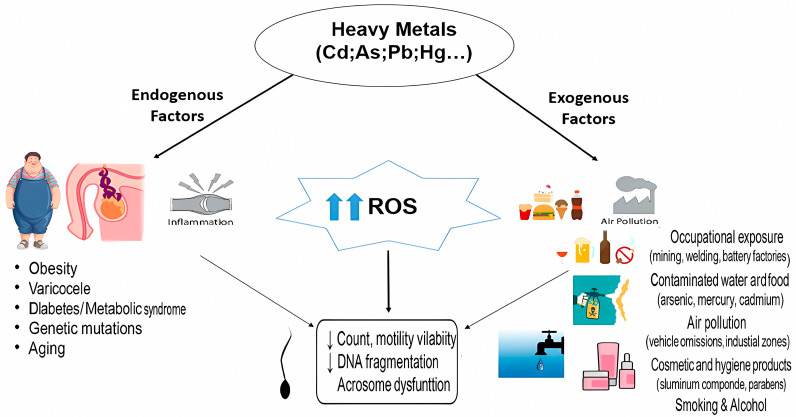
Presenting the relationship between heavy metal exposure and the excessive release of ROS, which influences ROS–antioxidant balance, resulting in significant changes in sperm parameters (mobility, vitality, morphology, and DNA integrity). All these factors contribute to male infertility.

Other studies mention other jobs in which production takes place, including those using pigments, hexamethylenetetramine, phenol, plasticizers, and benzene, and including chemistry workers and even laboratory technicians [17]. Recently in 2023, Bouchala et al. assessed the levels of cadmium, arsenic, and lead in 170 workers at a lead-acid battery manufacturing and recycling plant in eastern Algeria. Lead was the most present element in the blood of the workers (521.24 μg/L), showing that the conditions led to significant exposure to lead and possibly other elements such as Cd and As [18]. Heavy metals are also present in drinking water, such as lead and mercury from the atmosphere due to pollution from industrial activities which can be deposited in the soil around a reservoir and then enter the water with runoff from the surface [19]. Pesticides are also made up of several types of heavy metals that can be absorbed through the skin or inhalation. Male fertility has been demonstrated because of pesticides adopted and considered as potent endocrine disruptors [20]. Some evidence suggests that pesticides can influence spermatogenesis [21]. Personal care products include all non-pharmaceutical products present in their ingredients, such as phthalate esters, parabens, ultraviolet (UV) filters, and polycyclic masks [22]. These components can decrease reproductive capacity through damage to the testicular tissue or through endocrine disruption [23]. Heavy metals can disrupt the metabolic functions of the human body through a variety of pathways [24] (Figure 1).

### 2.1. Cadmium (Cd)

Cadmium is a divalent heavy metal (Cd^2+^), often released into the environment through industrial emissions, battery production, and phosphate fertilizers. Primary sources include occupational exposure, cigarette smoke, contaminated food, and polluted water [25].

Cadmium can affect the human reproductive system since it accumulates easily in tissues and organs, including testicles [26]. It induces an excessive release of reactive oxygen species (ROS). Furthermore, it can disrupt spermatogenesis by competing with essential elements such as magnesium, iron, zinc, selenium, and copper, resulting in a reduction in their concentrations. It is also associated with dystrophic calcification of testicular components, competing with calcium [27]. Recent studies have revealed that cadmium can trigger tissue inflammation by reducing the total amount of adiponectin [26]. Cadmium-mediated toxicity leads to a disturbance of the blood–testis barrier and alteration of seminiferous tubules [27]. Cadmium also causes significant damage to Sertoli cell DNA through increased apoptosis due to damage to cytoskeletal proteins such as actin and tubulin [28]. Cadmium can also harm Leydig cells as a source of androgens, contributing to the conservation of normal spermatogenesis [29]. Recent studies have shown that elevated cadmium concentrations in urine are associated with a decrease in the number of motile spermatozoa [30] and viability [31], while a high concentration of cadmium in the blood has been linked to an increased number of immotile spermatozoa and reduced sperm motility [32]. Furthermore, seminal plasma presents a concentration of some elements and has been demonstrated as a biomarker of the exposure status of the male reproductive system [33]. Higher concentrations of cadmium in seminal plasma affect sperm quality parameters (sperm count, motility, vitality, and morphology) [34,35].

### 2.2. Lead (Pb)

Lead is commonly found as Pb^2+^ and accumulates in bone and soft tissues, including testicular tissue. Occupational sources in professions such as battery recycling, construction, and painting are mainly through contaminated water and lead dust or soil [36].

Research on a murine model exposed to lead has demonstrated that this metal induces a series of injuries, smaller testicles, and deviant spermiogenesis. The acetylation of lysine can subsequently hinder the replacement of transition proteins in elongating sperm, resulting in the aberrant configuration of germ cells within the seminiferous tubule. The inhibition of Lysine acetylation occurs in meiotic spermatocytes and round spermatids, particularly during meiosis [37]. Marzec-Wróblewska and colleagues have shown a positive correlation between lead in seminal plasma and progressive sperm motility [38]. The impairment of spermatogenesis due to lead exposure may also result from an overproduction of reactive oxygen species (ROS) accompanied by the inhibition of and reduction in antioxidant enzyme activity. Lead induces testicular toxicity and thus on reproductive hormone levels and their receptors, causing disruptions in the hypothalamus–pituitary–testicle axis [39]. In vitro studies on mouse Leydig cell lines have shown that lead exposure can decrease cell viability and lead to morphological changes and apoptosis [40]. Recent studies have reported that lead-treated rats exhibited a significant reduction in sperm quality, a decrease in testicle and accessory sexual organ weights, as well as a decrease in testicular steroidogenic enzyme levels and serum testosterone [41].

### 2.3. Mercury (Hg)

Mercury exists in several forms, including elemental (Hg^0^), inorganic (Hg^2+^), and organic (CH_3_Hg^+^), with organic forms being the most toxic. Dietary intake, especially fish and seafood, are most sources of mercury liberation, but there can also be occupational exposure in manufacturing and from environmental contamination [42,43]. The level of mercury exposure is linked to its presence in the environment and various food products, potentially resulting in health consequences [44].

In male rats, exposure to mercury has adverse effects on reproductive characteristics, including impairments in spermatogenesis, reductions in sperm motility, and an increase in pathological changes [45]. Exposure to Hg^2+^ (ranging from 5 to 500 ng HgCl_2_) in humans and other male animals can alter spermatogenesis and semen quality [46]. Both rats and humans being exposed to HgCl_2_ leads to a decline in sperm concentration, sperm motility, and ejaculated semen volume, as well as the increase in sperm abnormalities and a decrease in testis weight [47,48]. In adult rats, the exposure of Hg^2+^ causes sperm DNA strand break through the production of reactive oxygen species (ROS) and disruptions in the antioxidant mechanism. In monkeys, exposure to CH_3_Hg^+^ (25 μg/kg/day for 20 weeks) has been demonstrated to decrease sperm count and motility and testosterone levels, increase sperm abnormalities, and damage testicular structure and Sertoli cells [49]. Some studies involving humans have shown that exposure to CH_3_Hg^+^ or Hg^2+^ leads to poor semen quality, primarily affecting sperm count and mobility [50]. 

### 2.4. Arsenic (As)

Arsenic is found in trivalent (As^3+^) and pentavalent (As^5+^) forms, and both of them are toxic to reproductive tissues. Arsenic exposure occurs through contaminated drinking water, particularly in some regions of Asia and South America; industrial processes; pesticides; and certain foods [51,52].

Spermatogenesis and histopathological changes in the epididymis in rats were observed during prepubertal exposure to doses of sodium arsenite, increasing also the abnormal configuration of sperm and levels of lipid peroxidation [53]. This agent induces a mitochondrial oxidative deficiency in the testicle and thus an accumulation of reactive oxygen species (ROS) and lipid peroxidation products, leading to Sertoli cells metabolic disruption [54]. There is a relationship between a high urinary arsenic concentration and a risk of abnormal progressive motility [35] and a low sperm count [31]. Some studies affirmed that concentrations of arsenic in seminal plasma affect progressive motility as well as sperm velocity. Wan et al. reported that arsenic exposure can also increase sperm DNA fragmentation [55].

### 2.5. Cobalt (Co)

Cobalt is a trace element essential in small amounts but toxic in excess, often found as Co^2+^ in ionic form. Different sources of exposure are industrial activities like metal processing, hard metal tools, orthopedic implants, and contaminated water or air [56].

Cobalt is a relatively rare natural element in the Earth’s crust. It circulates near the surface through various natural processes and anthropogenic activities. Cobalt is an essential element for mammals, serving as a constituent of vitamin B12 [57]. The injection of an important dose of cobalt–chromium nanoparticles (CoCr) leads to a significant decrease in sperm parameters, especially motility, viability, and concentration, accompanied by defective sperm quality and pathological alterations in the testicles due to the induction of oxidative stress reactions [58]. Furthermore, the sperm production was significantly reduced in these male mice. It causes lots of side effects such as hypertrophy of interstitial Leydig cells, degenerative changes in spermatogonia cells, and necrotic alterations in the seminiferous tubules and interstitial tissue [59].

### 2.6. Aluminum (Al)

Aluminum is typically found in the trivalent form (Al^3+^) and is not essential for human physiology. Exposure sources are dietary intake such as processed foods, additives, cookware, antacids, and water treatment [60].

Aluminum is one of the heavy metals abundant in our environment. Its ingestion in excessive amounts can lead to its accumulation in some organs, damaging testicular tissues and leading to a decrease in testis volume. High aluminum concentration in human testicles, seminal plasma, urine, and blood has a negative role in Leydig and Sertoli cells as it can alter sperm quality, especially the motility and viability of spermatozoa. Several studies suggested that aluminum exposure causes adverse impacts on male reproduction [61].

Jamalan et al. demonstrated that heavy metals are the principal cause of male infertility through their effects on of sperm quality. Strong evidence confirms that male infertility for cases with metal exposure is mediated though various mechanisms, such as the production of reactive oxygen species (ROS) reducing sperm motility and the prevention of oxidative damage of the membrane from aluminum chloride (AlCl_3_), cadmium chloride (CdCl_2_), and lead chloride (PbCl_4_) [62].

Higher concentrations of AlCl_3_ induce the production of free-radical-mediated cytotoxicity, and it is toxic for the male reproductive system [63]. Previous studies have demonstrated that the treatment using aluminum can decrease ejaculate volume, sperm concentration, and sperm motility [64].

Several studies investigating the impact of heavy metals on male fertility vary widely in terms of experimental design, exposure levels, and biological models. Several studies simulated real-life human exposure scenarios by using environmentally relevant concentrations of metals, such as those found in contaminated water, air, or food. For instance, in the case of cadmium, research by Ramos-Treviño et al. demonstrated deleterious effects on sperm count and morphology even at low chronic doses, mimicking environmental pollution levels. Similarly, other studies evaluated the impact of lead exposure at doses consistent with occupational exposure, reporting decreased sperm motility, increased apoptosis, and hormonal imbalances [8,65]. Other studies, such as those involving mercury, arsenic, or even aluminum, also relied on concentrations that reflect chronic environmental exposure, making the findings more directly translatable to human health risk assessment [42,60].

Conversely, several investigations purposefully employed higher, supra-physiological doses of metals to rapidly elicit measurable testicular damage or to dissect specific molecular pathways. For example, the work of Yousef et al. on aluminum involved short-term exposure to elevated doses, enabling the characterization of oxidative damage, lipid peroxidation, and structural degeneration of testicular tissue [64]. While such doses exceed typical environmental exposure, they are instrumental in identifying threshold effects, understanding mechanisms of toxicity such as oxidative stress, endocrine disruption, and apoptosis [65,66].

The data presented in Table 1 summarizes the main reproductive toxicities associated with several heavy metals, including cadmium, lead, mercury, arsenic, aluminum, and cobalt. While all these elements share the ability to induce oxidative stress through excessive production of reactive oxygen species (ROS), their mechanisms of action and biological consequences vary in both intensity and specificity. They cause direct structural damage to testicular tissue, apoptosis in Sertoli cells, endocrine disruption, and lipid peroxidation, leading to disrupted spermatogenesis, decreased sperm count, and increased DNA fragmentation and abnormal morphology.

**Figure 2:**
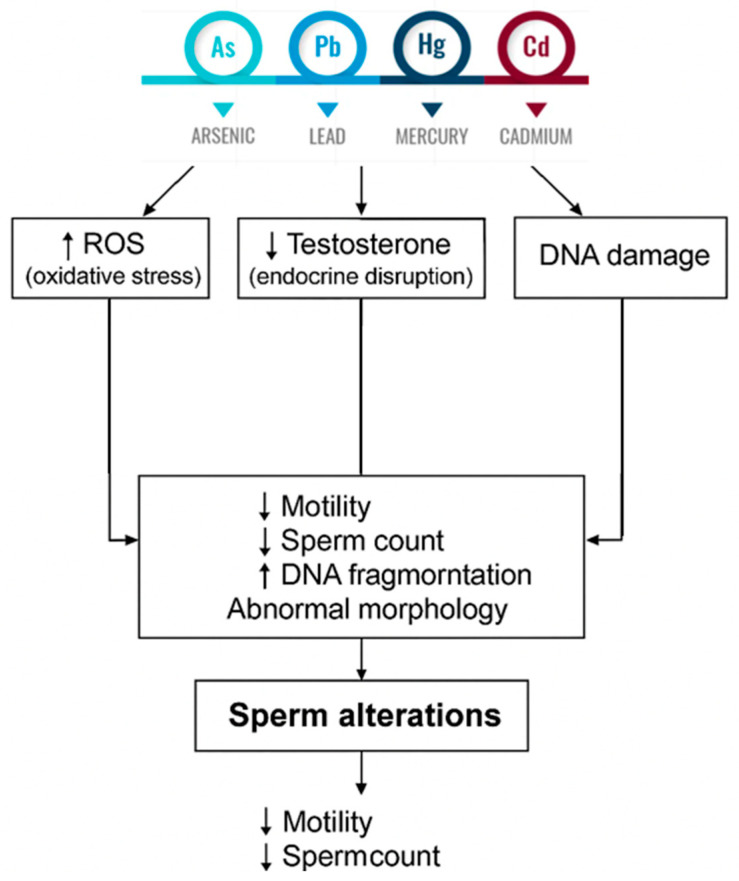
Summary diagram illustrating the main toxic actions of heavy metals on testicular cells and sperm function. Heavy metals induce oxidative stress (↑ ROS), apoptosis, and endocrine disruption (↓ testosterone). These disruptions lead to structural and functional sperm alterations, including decreased concentration, motility, and viability, abnormal morphology, acrosome dysfunction, and increased DNA fragmentation.

In summary, although heavy metals differ in their chemical properties, environmental sources, and routes of exposure, they converge on common pathophysiological mechanisms that compromise male fertility. These toxic agents induce significant oxidative stress, disrupt hormonal balance, impair the blood–testis barrier, and directly affect the viability and function of Sertoli and Leydig cells. The combined impact of these alterations leads to reduced spermatogenesis, decreased testicular volume, impaired sperm motility and morphology, and increased sperm DNA fragmentation. These effects, reported across various animal models and human studies, highlight the potentially harmful consequences of chronic exposure to heavy metal, even at low environmental levels and underscore the need for preventive and monitoring strategies in exposed populations. To provide a comprehensive overview of the testicular toxicity induced by heavy metals, a schematic summary (Figure 2) illustrating the main cellular targets and biological consequences associated with heavy metals exposure is provided. The figure highlights mechanisms of toxicity, the affected testicular structures, and the resulting alterations in sperm parameters such as reduced motility, abnormal morphology, and DNA fragmentation.

## 3. Oxidative Stress

Oxidative stress is characterized by an imbalance between the production of reactive oxygen species (ROS) and available antioxidants resulting in a redox paradox [70]. ROS are necessary for sperm maturation, acrosomal reaction, capacitation, hyperactivation, and sperm–oocyte fusion [71]. Among the endogenous origins of ROS is varicocele, which is a more common etiology of male infertility associated with increased oxidative load and ROS-induced sperm DNA damage with an abnormal increase in scrotal temperature [72]. Additionally, increased temperature has also been linked to highly increased ROS production and negative effects on other seminal parameters [73]. Elevated ROS levels are also associated with infections of male accessory glands, including the urethra, prostate, vas deferens, seminal vesicles, epididymis, or testes. A urogenital infection is characterized by the overproduction of leukocytes in the semen and their ability to produce 1000 times more ROS and free radicals than any other aerobically metabolic cells. A decline in sperm quality which could be responsible for the decline in the reproductive health of diabetic men [74]. Additionally, men with type 1 and type 2 diabetes have a disruption of the seminiferous tubule, erectile dysfunction, ejaculation dysfunction and an alteration on sperm parameters including sperm volume, number, motility, and morphology [75].

Cigarette smoking presents 48% in seminal white blood cell concentrations and a significant rise in seminal reactive oxygen species (ROS) levels, resulting in decreased antioxidant levels [76]. The consummation of alcohol induces oxidative stress as ethanol can stimulate the production of ROS [77]. A study involving 46 fertile-age alcoholic men suggested the presence of testicular oxidative stress by observing a marked decreased concentration of testosterone, an increase in serum lipid peroxidation, and a decline in antioxidants [78]. Several studies have demonstrated that DNA damage in sperm increases with advanced age among fertile men due to an imbalance in the ROS and antioxidant equilibrium [79].

**Figure 3:**
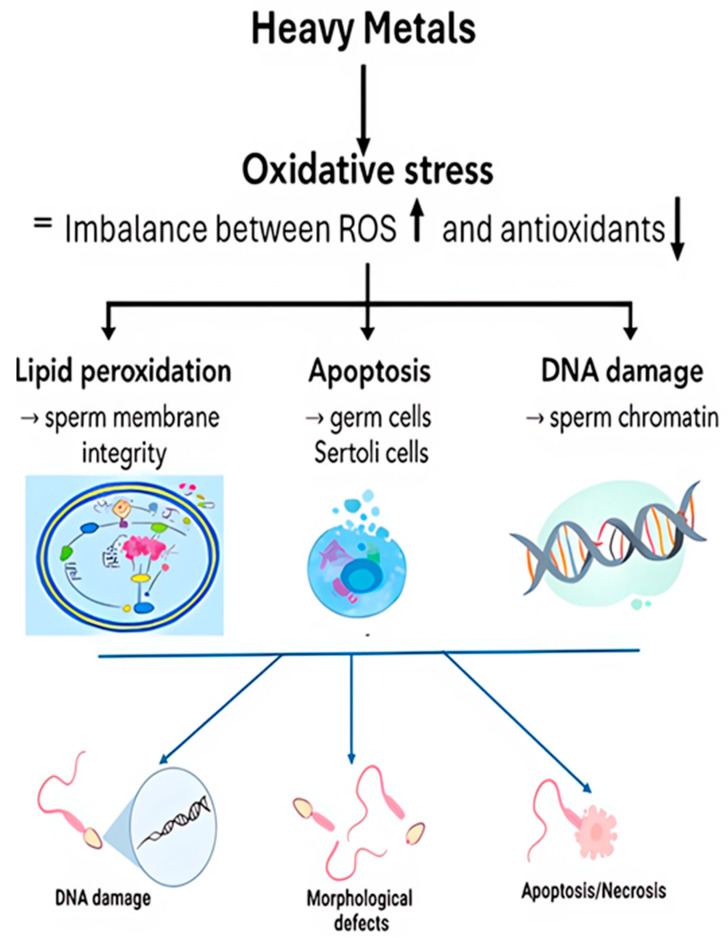
Presentation of oxidative stress triggered by factors that disrupt the balance between reactive oxygen species (ROS) production and antioxidant capacity in seminal plasma. The primary factors contributing to male infertility are the generation of harmful substances resulting from lipid peroxidation, the disruption of critical mechanisms leading to apoptosis, and DNA damage.

Oxidative damage is mediated by an overproduction of reactive oxygen species (ROS). When mammals are exposed to heavy metals, then we can have an over expression of ROS/RNS, for example, peroxyl radicals (ROO ^•^), superoxide radical, hydroxyl radical (OH ^•^), hydrogen peroxide and dimethylarsenic radical, and the peroxylated dimethylarsenic radical. The formation of oxidized lipids generates several bioactive molecules, especially aldehydes. Malondialdehyde (MDA) and 4-hydroxy-nonenal (HNE) are the main products and the increase in these free radicals will subsequently disrupt normal metabolism and the regulation of reproduction, especially for men. The increase in radicals will destroy spermatogenesis and reduce the level of testosterone released by Leydig cells [80] (Figure 3).

## 4. Antioxidants

### 4.1. Zinc (Zn)

Zinc is a vital trace element essential for normal spermatogenesis and steroidogenesis [81,82]. Zinc is essential for the maintenance of sperm chromatin stability, testosterone production, and the activity of antioxidant enzymes. Data has shown that males with asthenospermia/teratozoospermia have a notably lower zinc intake compared to fertile individuals [83]. Studies have also revealed a relationship between zinc concentrations in seminal plasma and sperm quality and testosterone levels in serum [84,85]. Depletion of zinc leads to a 50% reduction of zinc in the ejaculate, resulting in pathozoospermia [86]. In a clinical study involving 37 patients with unexplained infertility, supplementation with 24 mg of elemental zinc for 45 to 50 days lead to an increase in testosterone levels and sperm count, with counts rising from 8 to 20 million/mL and supporting 9 successful conceptions. Other studies have reported significant correlations between concentration of zinc in seminal plasma and sperm count, density, motility, and viability [87]. However, excessive zinc supplementation has been associated with reduced sperm motility, as described in one study in which there was a decline in human sperm motility in association with increased zinc levels [88]. In summary, zinc plays a significant role in male reproductive physiology.

### 4.2. Selenium (Se)

Selenium (Se) is an essential component found in numerous proteins known as selenoproteins. These proteins play pivotal roles in various metabolic pathways, antioxidant defense, redox regulation, and cancer prevention [89].

In the context of spermatogenesis, activation of mitochondria, and capacitation, selenium functions as a factor of antioxidative enzymes responsible for neutralizing and preventing the synthesis of reactive oxygen species (ROS) [86]. In spermatozoa it becomes part of the membrane of mitochondria and the ROS production that occurs during the motility [87]. Furthermore, selenoproteins are more expressed in the testicles and seminal plasma and are positively correlated with sperm concentration and viability [88]. Studies conducted by Mistry et al. demonstrated the positive effects of selenium supplementation on sperm parameters [89]. Additionally, research by Talebi et al. revealed that higher selenium intake can improve sperm total motility and ejaculated volume [90]. Excessive selenium intake may not yield therapeutic benefits and could potentially reduce male reproductive potential by disturbing the optimal balance of ROS required for motility and the acrosomal reaction [91]. In summary, when serum selenium levels are within appropriate ranges, sperm morphology remains of high quality [92].

### 4.3. Vitamin E

Vitamin E serves as the most important antioxidant within spermatozoa, safeguarding the plasma membranes of cells from the effects of reactive oxygen species (ROS) [93]. Research conducted by Rengaraj and Hong has shown that a deficiency in vitamin E can lead to abnormal spermatogenesis [94]. While vitamin E does have positive effects on testis and sperm functions, it is important to note that supplementation with vitamin E-containing prescriptions has limited impact on the overall sperm quality [93].

### 4.4. Vitamin C

Much like vitamin E, vitamin C plays a potential role in protecting cell membranes against ROS. In a study by Cyrus et al., the supplementation of vitamin C did not present any improvements in sperm concentration but did have some favorable effects on motility and morphology [95]. However, the actual effects of supplementation of this vitamin remain a subject of debate and require further investigation. Excessive intake may interfere with the physiological redox balance and sperm capacitation processes. Future clinical studies are required to study the importance of vitamin C in male infertility [96].

### 4.5. Vitamin B12

Vitamin B12, also known as cobalamin, acts as a factor in DNA integrity and plays an important role in the metabolism of fatty acids and amino acids. Vitamin B12 has a positive effect on sperm quality by improving sperm concentration and motility and reducing sperm DNA fragmentation [97].

Additionally, similar to alpha-lipoic acid (ALA), vitamin B12 has been studied for its ability to protect spermatozoa from damage during the freezing–thawing process used in Assisted Reproductive Technology (ART). Its inclusion in cryopreservation media enhances sperm viability and motility while reducing DNA fragmentation that can occur during freezing and thawing. In summary, compared to other antioxidants, only vitamin B12 has shown a significant role in managing subfertility through antioxidant supplementation. Moreover, prolonged intake of this vitamin may lead to alterations in methylation patterns, which could influence epigenetic regulation during spermatogenesis [98].

### 4.6. Coenzyme Q10

Coenzyme Q10 (CoQ10) is essential to produce energy and effectively safeguard cell membranes from damage caused by lipid peroxidation [99]. Within mitochondria, CoQ10 plays a crucial role in neutralizing ROS generated [100]. Infertile men often exhibit low levels of CoQ10 [101]. Consequently, there is evidence suggesting that CoQ10 can enhance sperm concentration and motility in infertile men [102]. Decreased levels of CoQ10 in seminal plasma can influence sperm parameters, notably motility, but an over administration of CoQ10 may alter mitochondrial membrane potential, which is critical for sperm motility.

Nadjarzadeh et al. conducted a double-blind, randomized clinical experience to investigate the supplementation of CoQ10 on seminal parameters in oligoasthenoteratozoospermic (OAT) men. They found a significant direct correlation between this supplementation and sperm motility and morphology. CoQ10 supplementation for three months mitigated oxidative stress in seminal plasma and enhanced antioxidant enzyme activity [103].

Similarly, García-Díaz et al. observed that three months of CoQ10 supplementation led to significant increases in sperm parameters (concentration, total and progressive motility) [104].

Thakur et al. found that the concentration of CoQ10, by bolstering the total antioxidant capacity, was related to semen parameters, including sperm concentration, motility, and morphology [105]. While the recommended dose of CoQ10 remains uncertain, it holds significant promise as a treatment option for idiopathic male infertility [101].

### 4.7. L-Acetylcarnitine

L-acetylcarnitine (ALC), an important antioxidant, can protect mitochondria from metabolic toxins. ALC also stabilizes cell membranes and causes anti-apoptotic actions [106]. L-carnitine is absorbed by epididymal cells and released into the epididymal lumen and luminal part of the seminiferous epithelium. The concentration of LC in the epididymis and in sperm cells presents approximately 2000-fold greater than in the circulating serum, implying it plays a very important role in sperm maturation, metabolism, and motility. As we know, the epididymis plays an essential role in sperm maturation, progressive motility, and the achievement of fertilization capacity by male germ cells [107]. Positive correlations between L-carnitine seminal levels with sperm count and motility have been demonstrated in different clinical studies. However, excessive intake may lead to the accumulation of acetyl-L-carnitine and may negatively affect Sertoli cells. In two studies which included 100 and 60 infertile men, respectively, sperm concentration and motility is shown to be increased. Similar results were found in another study which examined 60 men with oligo-asthenozoospermia [108]. In 52 men treated with clomifene, carnitine resulted in higher sperm concentration and motility [109].

## 5. Conclusions

Prolonged exposure to these heavy metals leads to chronic infertility issues, impacting the reproductive potential and breeding success of organisms. Comprehensive research into the sources and routes of exposure, the underlying mechanisms of action, and the adverse effect on human reproduction is essential to advance our understanding in this field. Given the well-documented impact of heavy metals on male fertility, preventive strategies are essential. Avoiding exposure to environmental and occupational sources of cadmium, lead, mercury, and arsenic is critical. This includes monitoring contaminated water, limiting consumption of affected seafood, and reinforcing protective measures in industrial settings. Furthermore, antioxidant supplementation has been investigated as a promising therapeutic strategy to counteract oxidative damage. Nutrients such as vitamin C, vitamin E, selenium, and zinc have shown potential to reduce ROS levels, protect sperm DNA, and improve semen parameters. It is noteworthy that oxidative stress-controlled reproductive infertility induced by heavy metals like Cd^2+^ and Pb^2+^ is reversible, and it can be mitigated through chelating agents and the relocation of individuals from environments laden with heavy metals. Further exploration of the therapeutic potential of natural antioxidants, such as vitamin C in lemon, vitamins C and E, N-acetyl-l-cysteine, and others, is warranted in the context of heavy metal toxicity. The male reproductive function is susceptible to various environmental and occupational hazards, although not all these compounds have been fully identified.

## Figures and Tables

**Table 1:** Summary table of the effect of various heavy metals on male fertility, sperm quality, and testicular volume.

Heavy Metals	Effect on Male Fertility	Sperm Parameter Affected	Mechanisms of Toxicity	References
Cadmium (Cd)	Destruction of spermatogenesis	Increase in motility Decrease in sperm count Decrease in sperm morphology	Increase in ROS Apoptosis in Sertoli cells	[8]
Lead (Pb)	Decrease in testicular volume	Decrease in progressive motility Increase in ROS Decrease in antioxidants Decrease in reproductive hormones Decrease in viability	Increase in apoptosis Endocrine disruption	[65]
Mercury (Hg)	Decrease in testis weight Decrease in testis structure	Decrease in total and progressive motility Decrease in semen quality and quantity Decrease in sperm volume	Increase in DNA damage Increase in ROS Lipid peroxidation	[42]
Arsenic (As)	Testicular degeneration	Decrease in sperm count	Increase in lipid peroxidation Increase in DNA damage Increase in ROS	[67]
Aluminum (Al)	Increase in testicular tissue damage Decrease in testis volume	Decrease in sperm viability	Decrease in sex hormones Increase in ROS	[64]
Cobalt (Co)	Decrease in testicular weight Spermatogenetic arrest	Decrease in sperm motility Decrease in sperm viability	Increase in ROS	[68,69]

ROS: reactive oxygen species.

## Data Availability

All the data included in this study can be obtained on request.
